# Numerical investigation of the effect of fluid pressurization rate on laboratory-scale injection-induced fault slip

**DOI:** 10.1038/s41598-023-30866-8

**Published:** 2023-03-17

**Authors:** Gergő András Hutka, Mauro Cacace, Hannes Hofmann, Arno Zang, Lei Wang, Yinlin Ji

**Affiliations:** 1 Section 4.8 Geoenergy, Helmholtz Centre Potsdam GFZ German Research Centre for Geosciences, 14473 Potsdam, Germany; 2 Institute for Applied Geosciences, Technical University of Berlin, 10587 Berlin, Germany; 3 Section 4.5 Basin Modelling, Helmholtz Centre Potsdam GFZ German Research Centre for Geosciences, 14473 Potsdam, Germany; 4 Section 2.6 Seismic Hazard and Risk Dynamics, Helmholtz Centre Potsdam GFZ German Research Centre for Geosciences, 14473 Potsdam, Germany; 5 Institute of Geosciences, University of Potsdam, 14476 Potsdam, Germany; 6 Section 4.2 Geomechanics and Scientific Drilling, Helmholtz Centre Potsdam GFZ German Research Centre for Geosciences, 14473 Potsdam, Germany

**Keywords:** Geophysics, Natural hazards, Seismology

## Abstract

The effect of normal stress variations on fault frictional strength has been extensively characterized in laboratory experiments and modelling studies based on a rate-and-state-dependent fault friction formalism. However, the role of pore pressure changes during injection-induced fault reactivation and associated frictional phenomena is still not well understood. We apply rate-and-state friction (RSF) theory in finite element models to investigate the effect of fluid pressurization rate on fault (re)activation and on the resulting frictional slip characteristics at the laboratory scale. We consider a stepwise injection scenario where each fluid injection cycle consists of a fluid pressurization phase followed by a constant fluid pressure phase. We first calibrate our model formulation to recently published laboratory results of injection-driven shear slip experiments. In a second stage, we perform a parametric study by varying fluid pressurization rates to cover a higher dimensional parameter space. We demonstrate that, for high permeability laboratory samples, the energy release rate associated with fault reactivation can be effectively controlled by a stepwise fluid injection scheme, i.e. by the applied fluid pressurization rate and the duration of the constant pressure phase between each successive fluid pressurization phase. We observe a gradual transition from fault creep to slow stick–slip as the fluid pressurization rate increases. Furthermore, computed peak velocities for an extended range of fluid pressurization rate scenarios (0.5 MPa/min to 10 MPa/min) indicate a non-linear (power-law) relationship between the imposed fluid pressurization rate and the peak slip velocities, and consequently with the energy release rate, for scenarios with a fluid pressurization rate higher than a critical value of 4 MPa/min. We also observe that higher pressurization rates cause a delay in the stress release by the fault. We therefore argue that by adopting a stepwise fluid injection scheme with lower fluid pressurization rates may provide the operator with a better control over potential induced seismicity. The implications for field-scale applications that we can derive from our study are limited by the high matrix and fault permeability of the selected sample and the direct hydraulic connection between the injection well and the fault, which may not necessarily represent the conditions typical for fracture dominated deep geothermal reservoirs. Nevertheless, our results can serve as a basis for further laboratory experiments and field-scale modelling studies focused on better understanding the impact of stepwise injection protocols on fluid injection-induced seismicity.

## Introduction

Hydraulic stimulation treatments are performed in geothermal projects to enhance the permeability of the targeted reservoir by fluid injection^1^. Fluid injection can be associated with an increased seismic hazard, especially for injection wells located in the vicinity of critically stressed faults, i.e. for faults that are stressed close to their static shear strength, and are favourably oriented with respect to the in-situ stress field^2^. Thus, hydraulic stimulation treatments must enhance reservoir permeability with minimum induced seismic hazard^3^. To achieve this, it is crucial to gain knowledge of the dynamics governing the frictional sliding of reactivated faults, induced by fluid injection^4–6^. It is a matter of debate whether magnitudes of the largest possible induced earthquakes can be effectively controlled and limited by tuning operational parameters during hydraulic stimulation treatments (e.g. total volume of injected fluid, well-head pressure, and injection rate)^7^, or if they are controlled primarily by the tectonic stress field and the local geology of the reservoir, as it is discussed for tectonic earthquakes^8^. Recent laboratory experiments and numerical studies have assessed the effects of pressurization rate on injection-induced seismicity^9–12^. These studies suggest that pressure-increase rates play an important, albeit not yet fully quantified, role in fault reactivation and the promotion of subsequent seismic/aseismic slip.

The goal of this study is to further examine the effect of imposed fluid pressurization rates on induced seismicity in laboratory-scale injection-driven shear slip tests using numerical simulations based on an RSF formalism. We consider a stepwise injection scenario in which each fluid injection cycle consists of a fluid pressurization phase and a subsequent constant fluid pressure phase. Our goal is to explore the transition characteristics of the injection-induced fault slip mode from fault creep towards slow stick–slip behaviour as a function of the imposed fluid pressurization rate. In particular, we focus our investigation on how the applied fluid pressurization rate affects the stress drop on the laboratory fault during an induced slow slip event. To achieve this goal, we first calibrate our model formulation to recently published laboratory results of injection-driven shear tests^11^. In a second stage, we perform a parametric study on the imposed fluid pressurization rate and selected model properties. Our study suggests that a deeper understanding of these processes could help to develop operational methods to better control injection-induced seismicity on pre-existing natural and induced faults.

## 2D model of the injection-driven shear tests on faults

We calibrate our model formulation to two injection-driven shear tests conducted by Wang et al. (2020a)^11^. In these experiments, two different stepwise fluid pressurization schemes were applied to homogeneous and isotropic Bentheim sandstone samples with a critically stressed sawcut fault to examine the effect of varying fluid pressurization rates on the induced fault slip behaviour. The initial porosity of the samples is ~ 23%, and their permeability is ~ 1 Darcy.

We note that the high porosity and permeability of the Bentheim sandstone may not be fully representative of typical conditions in enhanced geothermal reservoirs. However, it was chosen for the original experiments^11^ to ensure a relatively homogeneous pressure distribution across the fault surface during the fluid injection test. Assuming that the effective stress law holds irrespective of the rock type, fault reactivation should depend only on the evolution of the friction coefficient and the effective stress on the fault^13^. Although low permeability rocks are more representative of enhanced geothermal reservoirs, experiments performed on such rocks (e.g., granite) with fluid conduits through boreholes^10,12^ are more difficult to interpret, considering the competing propagation of both the pressure and rupture front under locally undrained conditions. Therefore, we consider the data from Wang et al.^11^ as the simplest but physically relevant input for our numerical study to gain a better understanding of injection-induced seismicity.

**Figure 1:**
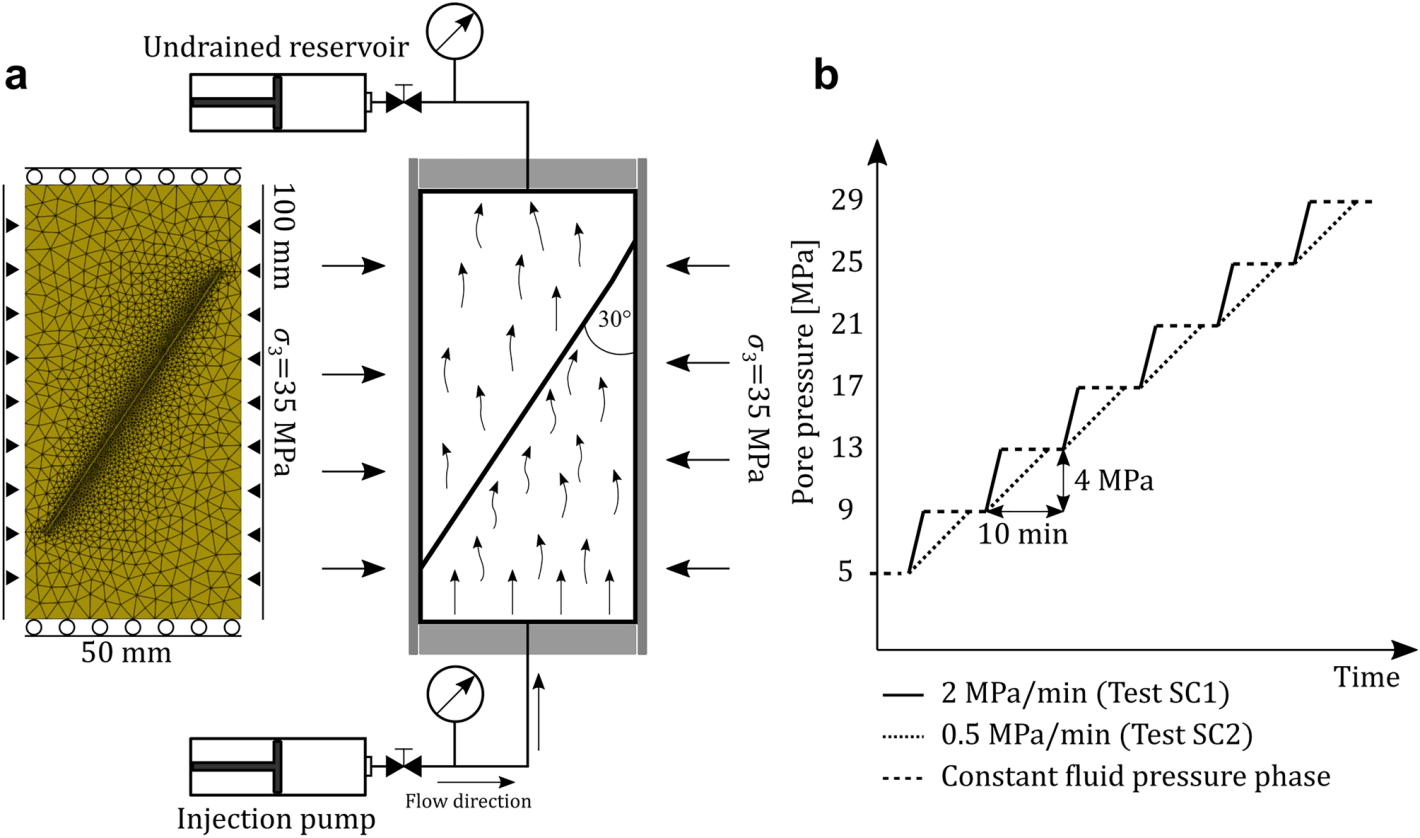
(**a**) Schematic figure of the original experimental setup and the model geometry with the mesh and boundary conditions adopted in the numerical study. (**b**) Pressurization schemes in test SC1 (2 MPa/min) and test SC2 (0.5 MPa/min).

In the original experiments, two cylindrical samples were prepared with a length of 100 mm and a diameter of 50 mm, each having a saw-cut fault oriented at approximately θ = 30° to the longitudinal axis of the sample, resulting in an elliptical fracture surface 50 mm along the minor axis and 100 mm along the major axis (Fig. 1a).

The samples were then loaded hydrostatically up to a targeted confining pressure of 35 MPa, while maintaining the pore pressure constant at 5 MPa. Next, the axial load was gradually increased with a displacement rate of 1 $\upmu \mathrm{m}/\mathrm{s}$ to reach the static shear strength of the fault (~ 38 MPa). The samples were then unloaded (with a displacement rate of 0.05 $\upmu \mathrm{m}/\mathrm{s}$) until the shear stress resolved on the fault was reduced to 92% of the nominal peak strength of the sample. From this point on, the position of the axial piston was maintained fixed during the subsequent fluid injection tests. The confining pressure was also kept constant at 35 MPa for the entire duration of the experiment.

Distilled water was then injected through the bottom surface of the sample, while the top of the sample was connected to a pump with a fixed piston to enforce undrained conditions. Fluid pressure was increased from 5 MPa up to a maximum of 29 MPa in a stepwise manner, divided into 6 cycles of 10-min. The pressure was increased by 4 MPa during each cycle. Two experiments were performed, which differed in terms of the imposed fluid pressurization rate: (i) in the first test, the 4 MPa pressure steps were achieved at a pressurization rate of 2 MPa/min—high fluid pressurization rate experiment (test SC1 hereafter); (ii) in the second test, performed on a separate sample, the pressurization rate was lowered to 0.5 MPa/min—low fluid pressurization rate experiment (test SC2 hereafter). After each pressurization phase, the fluid pressure was held constant for the remaining period of the 10-min cycle, which is referred to as the constant pressure phase, as opposed to the prior pressurization phase (Fig. 1b).

In Table 1 we list the material properties and the corresponding model parameters. The elastic and hydraulic properties of the rock matrix have been measured by Wang et al.^11^. No velocity stepping experiments were performed prior to the hydraulic experiments. Therefore, in order to derive a range of reasonable RSF parameters for the numerical model we relied on available experiments as those performed by Hunfeld et al.^14^ on other sandstone samples (Table 2). We used the maximum slip velocities measured in the laboratory as a metric for model calibration, which we consider a preferential indicator for the level of induced seismic hazard. In addition, slip velocity magnitudes provide an efficient way to determine the slip mode of the fault^15^. The RSF parameters providing the best fit for the slip velocities recorded in test SC1 are listed in Table 2. Note that we also use the same parameters for the simulation of test SC2. In Supplementary Fig. S1 we discuss the effect of varying the combined (a–b) parameter on the computed slip velocity, shear stress, and cumulative slip magnitudes.

**Table 1:** Material properties of Bentheim sandstone and the corresponding model parameters^11^.

Material property	Measured		Model	
	Domain	Fracture	Domain	Fracture
Bulk modulus (GPa)	13	–	13	13
Shear modulus (GPa)	11	–	11	10
Permeability (D)	1	–	1	10
Porosity (–)	0.23	–	0.23	1

**Table 2:** Rate-and-state parameters measured on Slochteren sandstone samples by Hunfeld et al.^14^ and the parameter values used in our model.

RSF fracture property	Hunfeld et al.^14^	Numerical model
Static friction coefficient (–)	–	0.6
a (−)	[3e-3; 6e-2]	1.25e-2
b (−)	[8e-4; 5e-2]	1.5e-2
(a–b) (−)	[− 6e-5; 5e-3]	− 2.5e-3
${D}_{c}$ (m)	[2e-6; 3e-4]	1e-5

The combined (a–b) parameter determines whether a fault governed by a rate-and-state formalism is in a regime of rate strengthening or rate weakening, the former characterised by positive values and the latter by negative values of (a–b). The value of (a-b) was chosen in agreement with the available experimental data, which indicate that sandstones behave as slightly rate weakening materials. This is a necessary, though not exclusive, condition for the development of stick–slip instabilities as observed in both laboratory and numerical experiments, at least at high fluid pressurization rates^16^.

It is worth noticing that in our modelling approach we downsize the model dimension to a 2D case with a one-dimensional saw-cut fault (Fig. 1). The fault is defined as 1D interfacial discontinuous element embedded in the 2D matrix, discretized by overlapping edges from the adjacent 2D elements. In our model, we limit the lateral extent of the fault so that it does not reach the model boundaries. This was done since it provides a computationally efficient, more stable solution to model the confinement to the sample in terms of pressure loading boundary conditions only along the lateral boundaries as done in the original experiments without imposing any constraints on the degrees of freedom for the fault deformation (similar to classical adopted free slipping boundary conditions). The initial pre-stressing phase is simulated by controlling the vertical displacement of the top model boundary while the bottom boundary is fixed, then we impose zero-displacement boundary conditions along the top and bottom boundaries to match the boundary conditions of the real experiment. Fluid injection is simulated by imposing the injection pressure-history derived from the laboratory experiments on the bottom boundary of the domain. The high permeability of the Bentheim sandstone allows for rapid fluid diffusion, leading to a homogeneous pressure distribution within the sample.

## Results

### Model calibration

During model calibration we aimed to match the maximum slip velocities of the 2 MPa/min fluid pressurization rate scenario (test SC1), as the injection-induced slip velocity is a good indicator of the induced seismic hazard^15^. This assumption is supported by the cumulative number of acoustic events recorded in the laboratory, which positively correlates with measured slip rates^17^.

Based on the peak velocities recorded in the two laboratory tests, we describe the observed aseismic slip events as fault creep when characterised by peak slip velocities < 1 µm/s and a longer duration of slip, and as slow stick–slip events for peak slip velocities < 1 mm/s (1 mm/s is considered the lower threshold velocity for seismic slip events) and a quasi-instantaneous slip compared to fault creep^11,18^.

**Figure 2:**
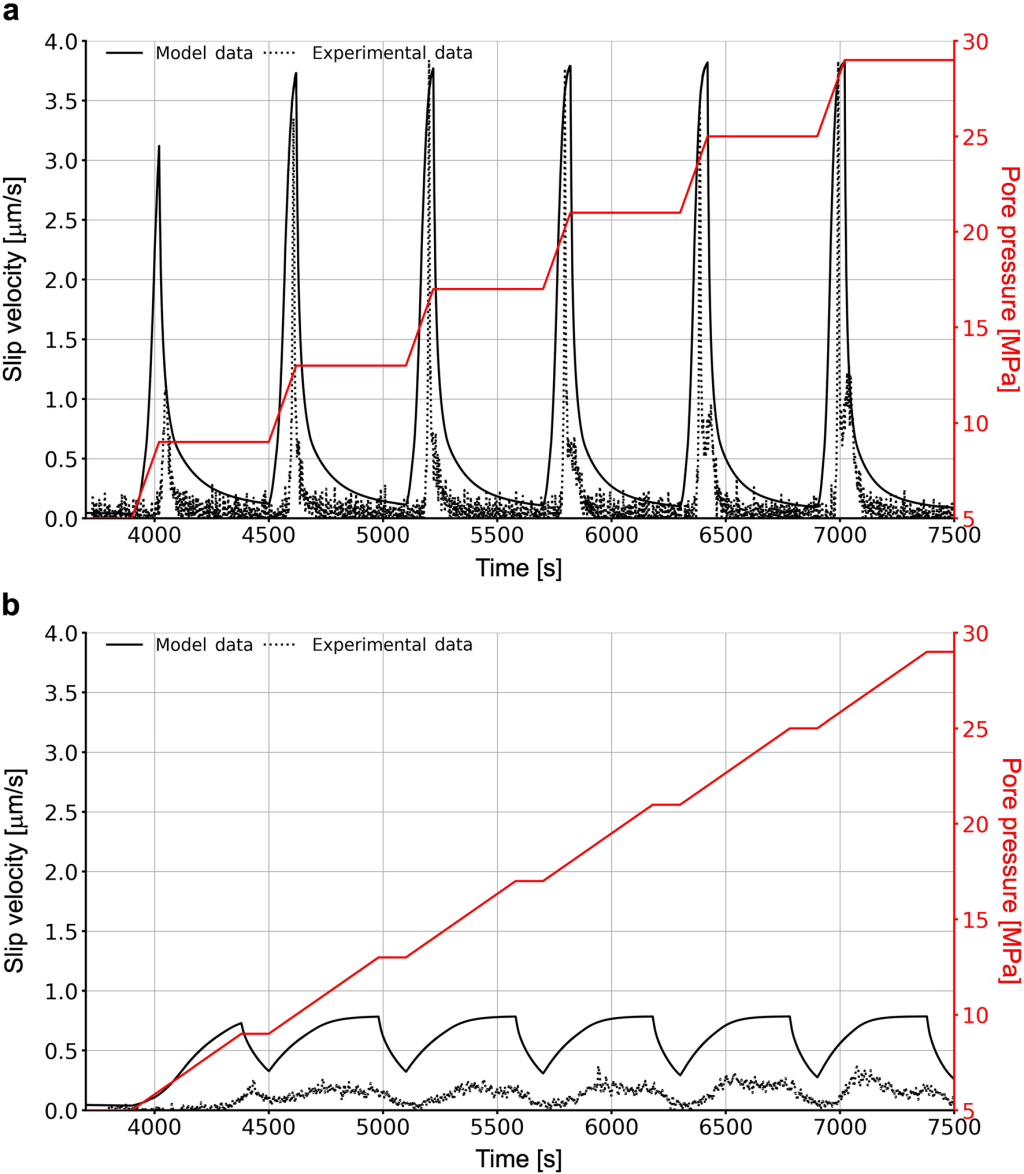
Measured and simulated slip velocity (**a**) for test SC1 with a pressurization rate of 2 MPa/min; (**b**) for test SC2 with a pressurization rate of 0.5 MPa/min.

**Figure 3:**
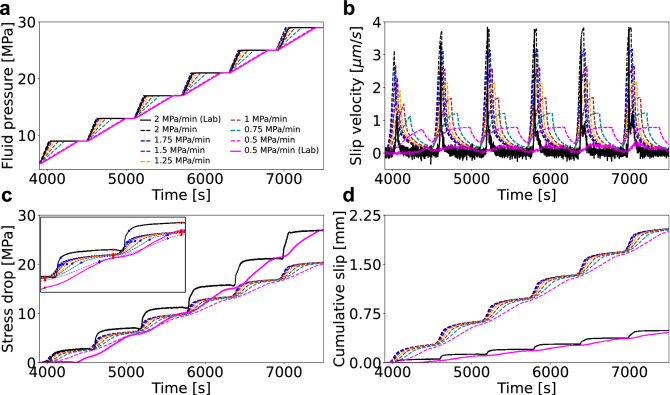
Parametric study on the imposed fluid pressurization rates. (**a**) Pressurization schemes; (**b**) slip velocity; (**c**) shear stress drop resolved on the fault; (**d**) cumulative slip. Dashed lines: simulated curves; solid lines: laboratory measurements. The inset in panel c shows a close-up of the simulated stress drop in two consecutive cycles. The red and blue dots mark the beginning and end of a given pressurization phase, respectively.

Computed peak slip velocities are in good agreement with the experimental data recorded for test SC1 (Fig. 2a). The higher fluid pressurization rate in test SC1 induces slow stick–slip events with peak velocities around 3.5 µm/s during the active pressurization phase, followed by decaying slip rates during the constant pressure phase. Slip events induced by the lower fluid pressurization rate of 0.5 MPa/min in test SC2 are still episodic, however, without the velocity spikes observed in test SC1 and span a longer time window. In our model of test SC2, computed slip velocity values are about three times as high as those measured in the laboratory. Nonetheless, the transition from a slow stick–slip behaviour towards a fault creep regime by decreasing the fluid pressurization rate is well captured by our results. While the maximum slip velocities in test SC1 are matched well with our model, the simulated peak velocities in test SC2 are about three times higher than in the experiment. Furthermore, the shear stress and cumulative slip histories do not match quantitatively in either case (Fig. 3c,d). The characteristics of the simulated velocities also differ from what we observe in the experiments. In contrast to the short duration velocity pulses recorded during the 2 MPa/min laboratory test, our simulation results show longer periods of increased slip velocities, which explains the higher cumulative slip found in our modelling results. Despite these differences, the varying trend in the shear stress and displacement is captured by the modelling, with lower pressurization rates resulting in a more continuous evolution of shear stress and cumulative slip, indicating preferential fault creep (Fig. 3b–d). Note that despite the magnitude offset relative to the laboratory data, we find that the final cumulative slip is not affected by the applied rate of fluid pressurization (Fig. 3d), as observed in the laboratory experiments. Potential reasons for the discrepancies between the laboratory data and the modelling results are explained in more detail in the discussion.

### Parametric study

Based on the calibrated model we carried out a detailed parametric study by gradually varying the applied fluid pressurization rate between a maximum of 2 MPa/min and a minimum of 0.5 MPa/min in steps of 0.25 MPa/min (Fig. 3a). This was done in order to resolve the details of the transition between the slow stick–slip and fault creep behaviour observed at higher (test SC1; Fig. 2a) and lower fluid pressurization rates (in test SC2; Fig. 2b), respectively.

The modelling results do not exhibit a sharp transition in velocity marking the onset of slow stick–slip. Instead, we observe a gradual transition in fault behaviour through the evolution of slip velocity (Fig. 3b), stress drop (Fig. 3c) and cumulative slip (Fig. 3d).

**Figure 4:**
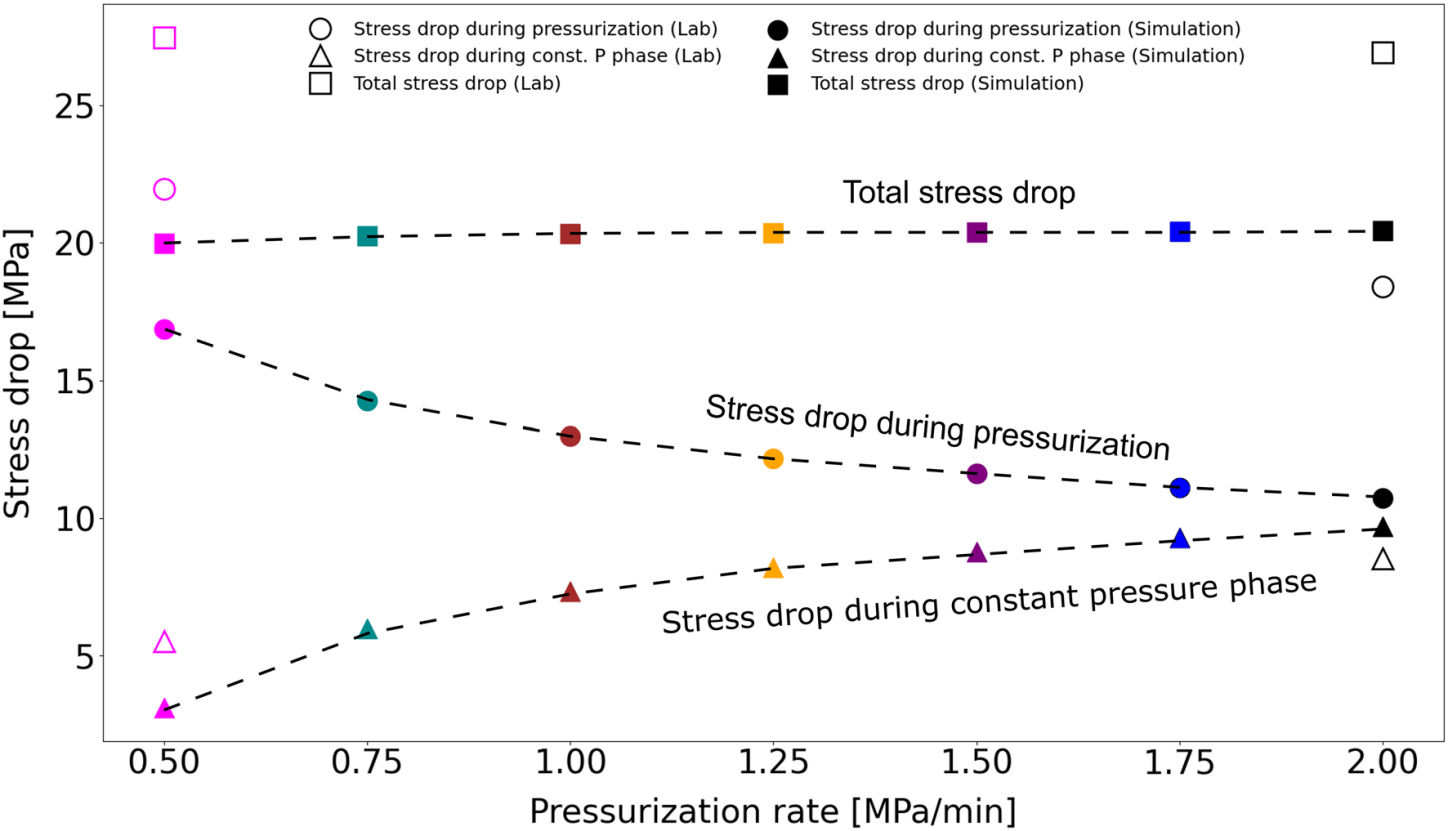
The computed effect (solid symbols) of the applied pressurization rate on the shear stress drop summed over each pressurization phase (circles), over each constant pressure phase (triangles); and the total stress drop (squares). Laboratory values are represented by open symbols. The colours represent different pressurization rates and remain the same as in Fig. 3.

In Fig. 4 we plot the resulting shear stress drop as a function of the imposed fluid pressurization rates both for the laboratory experiments and the models. In addition to the total stress drop, computed as the difference of the shear stress resolved onto the fracture at the beginning and at the end of the fluid injection test, we also calculate the stress drop that occurs during active pressurization and constant pressure phases over the course of the whole test, separately. We first compute stress drop values for a single injection cycle. During pressurization, this equals to the difference between the shear stress value computed at the beginning of the cycle when the pressurization starts (red dot on the shear stress curve in Fig. 3c) and the shear stress value corresponding to the termination of fluid pressurization (blue dot on the same shear stress curve in Fig. 3c). This is then repeated for all the six cycles. The resulting values are finally summed up to obtain the total stress drop during pressurization for the whole experiment or simulation. The total stress drop under constant pressure is calculated in a similar manner. The difference is that stress drop during the constant pressure phase of a single cycle is calculated by subtracting the shear stress value at the end of the constant pressure phase (i.e. at the end of the cycle; red dots in Fig. 3c) from the shear stress value at the end of the pressurization phase (blue dots in Fig. 3c).

In agreement with the laboratory experiments, we found that the computed total stress drop, given by the sum of the two contributions, is independent of the applied fluid pressurization rate (solid squares in Fig. 4). This observation is confirmed by an additional set of simulations for the same pressurization rate scenarios as in the original parametric study but including only a single fluid pressurization phase (increasing the pressure from 5 to 9 MPa), followed by a long constant pressure phase, allowing the fault to creep until the end of the simulation (Fig. S9). While the modelled total stress drop and its contribution from the pressurization phase differ in magnitudes with respect to their experimental values (that show ~ 7 MPa higher total stress drop), the computed stress drop during the constant pressure phase (solid triangles in Fig. 4) is in good agreement with the laboratory data. We notice that the higher the fluid pressurization rate, the larger the contribution from the constant pressure phase to the total stress drop, and that it increases as a non-linear function of the imposed pressurization rate. Further laboratory experiments with different pressurization rates are required to resolve and confirm the non-linear trend observed in the numerical model results.

**Figure 5:**
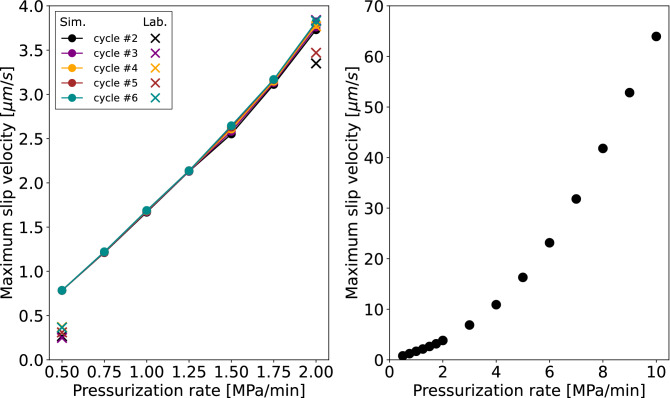
Computed maximum slip velocities versus pressurization rates (solid circles); (**a**) between 0.5 and 2 MPa/min for stimulation cycles 2 to 6. The first cycle was omitted due to its outlier values (fluid injection stopped shortly after the onset of fault reactivation); (**b**) for fluid pressurization rates ranging from 0.5 to 10 MPa/min. Laboratory values are denoted by crosses at 0.5 MPa/min and 2 MPa/min.

In Fig. 5a, we plot the computed maximum slip velocities as a function of applied fluid pressurization rates between 0.5 and 2 MPa/min for cycles 2–6. Cycle 1 is not shown because in test SC1 the fluid injection stopped shortly after the onset of fault reactivation, resulting in outlying maximum slip velocities. Between 0.5 and 2 MPa/min we observe a linear relationship with the resulting peak slip velocities, with a slight deviation from linearity above 1.25 MPa/min. We extended the range of tested fluid pressurization rates up to 10 MPa/min which revealed a non-linear relationship, which becomes clearly visible above ~ 4 MPa/min (Fig. 5b).

It is important to note that the duration of the constant pressure phase is directly proportional to the fluid pressurization rate, given that the pressurization cycle duration is kept constant at 10 minutes in all cases. Therefore, lower fluid pressurization rates result in shorter constant pressure phases, while higher fluid pressurization rates result in longer constant pressure phases. For this reason, we performed additional numerical simulations for all pressurization rates with a fixed, 480 s long constant pressure phase (Supplementary Figs. S2–S4). The results from this additional analysis do not differ significantly from the original observations in Fig. 3, 4 and 5, indicating that the length of the constant pressure phase (at least within the range of 120–480 s) does not have a significant influence on our findings.

In order to confirm the observations shown in Fig. 5, further laboratory experiments need to be conducted at intermediate (i.e. between 0.5 and 2 MPa/min) and higher (i.e. > 2 MPa/min) fluid pressurization rates. The tests carried out at a fluid pressurization rate of 0.5–2 MPa/min represent two end-member cases in terms of induced frictional behaviour, i.e. the slow stick–slip behaviour at 2 MPa/min and the fault creep at 0.5 MPa/min fluid pressurization rate, which we therefore consider representative, although not exhaustive, to capture the whole parameter space in the system response to imposed pressurization rates.

## Discussion

We simulated two injection-induced shear tests performed on high-permeability Bentheim sandstone samples intersected by saw-cut faults and compared our models with available laboratory results^11^. The peak values of the induced slip velocities in the model with the highest fluid pressurization rate (2 MPa/min; test SC1) are in good agreement with the laboratory data (Fig. 2). In contrast, our simulation of the test with a lower fluid pressurization rate (0.5 MPa/min; test SC2) and the same rate-and-state parameters resulted in peak velocities three times higher than in the experiment. We note that our model’s frictional parameters are calibrated based on the results of test SC1 alone. Therefore, despite similar samples, it is likely that the two model realizations require a different set of frictional parameters to fully match the magnitudes observed in the laboratory. Furthermore, it should be noted that our model is based on a rate-and-state formalism, which is still an empirical approximation to the actual frictional behaviour of real faults. This challenges the validity of a rate-and-state model to capture the mechanism of fluid injection-induced fault reactivation and highlights the need to develop novel physically driven and thermodynamically consistent models to improve the quantitative description of the frictional response of reactivating faults to fluid injection. Despite its limitations, the model shows that the slip induced at low fluid pressurization rates exhibits the characteristics of fault creep rather than the slow stick-slip behavior seen in the higher rate scenario, which is in agreement with the laboratory data. Therefore, we conclude that our model is able to capture the main macroscopic features of the experiments, i.e. the transition between a creep and a slow stick–slip behaviour controlled by the applied fluid pressurization rate. Regardless of the quantitative match in peak velocities of the high fluid pressurization rate experiment, we also note differences between our numerical model and this laboratory test. The model reproducing the laboratory results from test SC1 portrays slip events that start simultaneously with the onset of fluid pressurization at the beginning of each cycle. This differs from what is observed in the original laboratory data, where we see a systematic delay in the onset of slip after fluid pressurization starts (~ 60 s in the 2 MPa/min scenario and ~ 30 s in the 0.5 MPa/min scenario). In a similar fashion, during the decelerating slip phase towards the end of the pressurization phase, the decline in the slip velocity is also more instantaneous in the experiment than in the models. These observations may be related to the differences in the slip and shear stress histories displayed in Fig. 3, where the laboratory data show a larger stress drop and lower cumulative slip for all cycles. A possible reason for these discrepancies is that the effect of the MTS machine stiffness is not considered in our model. The stiffness of the sawcut fault is ~ 60 MPa/mm, while the machine has a stiffness of ~ 330 MPa/mm. As also described by Wang et al.^17^, a fast fluid pressurization of the sample likely leads to a rapid decrease in the fault frictional strength exceeding the response time of the loading system. This then accelerates the stress relaxation of the loaded system-sample assembly (which is not captured by our model), thereby resulting in a larger stress drop than the one modelled.

It is worth noting, that laboratory and modelled stress drop values are matched relatively well for the constant pressure phase. One potential reason for this is that in the constant pressure phase, the stress relaxation of the sample occurs much slower than in the pressurization phase, so the MTS machine has sufficient time to respond to the deformation of the system-sample assembly, unlike in the pressurization phase.

In addition, the model is also limited in describing the dynamics of the injection-induced fault slip of the laboratory experiments given our assumption of constant hydraulic properties and, most importantly to the case at hand, constant rate-and-state parameters. A previous modelling study by Yang et al.^19^ demonstrated that for cases in which the temporal variation of porosity and permeability (i.e. the shear-induced dilation or compaction of the fault) is not coupled to the rate-and-state controlled aseismic slip, the onset of the computed slip is delayed and the final cumulative slip is likely to be overpredicted, as it is the case in our models.

A functional behaviour of the combined (a–b) parameter with respect to pore pressure^20^ or slip velocity^21^ has been considered as a potential mechanism to explain the transition from fast to slow earthquakes in subduction settings, where the asperities within a megathrust front gradually move towards a rate strengthening regime over time as pressure or slip evolve. However, there is no direct evidence for such behaviour, as different laboratory studies give different, sometimes even contradictory results^22,23^. Linker and Dieterich^24^ discussed how the rate of change in the normal stress acting on the fault (stressing rate term) can additionally influence the fault frictional behaviour. Following these studies, we additionally tested our model by integrating the stressing rate term following the formalism by Nagata et al.^25^ (Fig. S8). The inclusion of this additional term only leads to a slight change in the system response, but does not change the cumulative behaviour. The laboratory observations and modeling results are consistent in showing a constant total stress drop. Despite the disparities between our model and the experiments, the fluid pressurization rates imposed appear to affect the macroscopic mode of energy release (and consequently the slip velocity magnitudes), but not the resulting slip and stress drop, which aligns with the findings of prior theoretical modeling studies (e.g. Fan et al. 2014)^26^.

To gain a better insight into how the rate of pressure-increase can affect the mode of the induced fault slip, we performed a numerical parameter study by varying fluid pressurization rates. Our results indicate a gradual transition from fault creep towards slow stick–slip with increasing fluid pressurization rate (from 0.5 MPa/min to 2 MPa/min), rather than an abrupt change in the sliding mode. We additionally found that with lower fluid pressurization rates, a larger portion of the total stress drop occurs during the active pressurization phase. Furthermore, a lower fluid pressurization rate decreases the maximum and average slip velocities in the pressurization phase, and leads to a slight increase of the minimum slip velocity in the constant pressure phase. Overall, both the maximum slip velocity and the difference between slip velocities during pressurization and constant pressure phases are lower for lower fluid pressurization rates. A non-linear correlation between slip velocity magnitudes and pressurization rate implies a faster drop in the frictional strength for higher pressurization rates and therefore a higher probability that the finite-size instability would trigger a run-away rupture. Run-away instabilities are hindered in our study that is based on a finite sample size, which, given the hydromechanical property of the rock investigated, is smaller than the critical nucleation length. Therefore, further reservoir-scale simulations are required to upscale these results to real case conditions.

The conclusions derived in this study are valid for the particular rock investigated, a porous and permeable Bentheim sandstone. Low permeability crystalline rocks may exhibit a different behaviour during a similar fluid injection test. Ji & Wu^12^ performed a series of injection-driven shear tests on sawcut faults in low-porosity (0.26%) granite samples. They showed that in the low-permeability (~ 1.3 μD) rock, fluid injection causes a heterogeneous pressure distribution in the fault, strongly influencing its frictional stability. Specifically, the rupture front propagates beyond the pressurized area of the fault, in contrast with the experiments performed on Bentheim sandstone, where the rupture front is constrained within the pressurized zone due to the high permeability (~ 1 D) of the sample^17^. The difference in the fluid pressure distribution on the fault associated with fluid injection is mainly attributed to the permeability contrast between high-permeability sandstone and low-permeability granite. This may lead to differences in the amount of elastic strain energy (indicated by the stress drop) releasing during the pressurization and constant pressure phases on the fault subject to stepwise increasing fluid pressure. To gain insight into the role of rock matrix permeability in delaying the stress drop, we conducted an additional set of simulations assuming a low permeability matrix (10^–6^ D); see Supplementary Fig. S5–S7). For the imposed fluid pressurization rates ranging from 0.5 MPa/min to 2 MPa/min, the fault exhibits creep behaviour in all cases, with comparable peak slip velocities (Supplementary Fig. S7). This could be explained by the presence of the low-permeability rock matrix that constrains the fluid pressure build-up only to the vicinity of the lower boundary, where the pressure is imposed. Despite this change in the fault behaviour, we still observe the same trend in the amount of stress drop occurring during the active pressurization phase and the following constant pressure phase (Supplementary Fig. S6).

The simulated experiment is analogous to a step rate test featured by stepwise increasing fluid pressure. Our results suggest that provided a high permeability reservoir and a direct hydraulic connection between the injection well and a high-permeability fault, by choosing the pressure steps and the fluid pressurization rates carefully, operators might be able to exercise some degree of control over the maximum slip velocity of the fault, as well as the slip and stress drop distribution between pressurization and constant pressure phases, while the total fault or fracture displacement remains the same. The permeability of self-propped, displaced fractures is found to be correlated to the resolved shear displacement along the same fault^27^ (although with a certain slip rate dependency^28^). Our results therefore suggest that it is theoretically possible to achieve the same permeability enhancement (i.e., shear displacement) with lower seismic hazard by imposing lower fluid pressurization rates. The applicability of our findings to deep geothermal reservoirs and EGS systems is limited by the difficulty of upscaling the conditions studied in the laboratory to field scale conditions. Therefore, further numerical investigations are needed to determine the potential practical utility of our results.

## Methods

### Fault slip, slip velocity and shear stress

In the experiment, fault slip displacement, $s$, is computed by projecting the net axial displacement that is determined from the total axial displacement (measured by an external LVDT), $\Delta {l}_{LVDT}$, minus the axial shortening of the loading apparatus, $\Delta {l}_{MTS}$, and the rock matrix, $\Delta {l}_{RM}$, as:

1 $$s=\frac{{(\Delta l}_{LVDT}-{\Delta l}_{MTS}-{\mathrm{e}l}_{RM})}{\mathrm{cos\theta }}$$

where $\uptheta$ is the angle between the fault plane and the long axis of the sample (30°). The slip rate is the time derivative of the fault slip displacement, $s$. The shear stress, $\tau$, and effective normal stress, ${\sigma }_{N}^{^{\prime}}$, resolved on the fault plane are calculated from the measured axial stress, ${\sigma }_{1}$, confining pressure, ${\sigma }_{3}$, and pore pressure, ${P}_{p}$, as:

2 $$\tau =\left({\sigma }_{1}-{\sigma }_{3}\right)sin\mathrm{\theta cos\theta }$$

3 $${\sigma }_{N}^{^{\prime}}=\left({\sigma }_{3}-{P}_{p}\right)+\left({\sigma }_{1}-{\sigma }_{3}\right){\mathrm{sin}}^{2}\theta$$

The shear strength of the fault is determined by plotting the shear stress as a function of slip displacement. After the sample yields, the plateau of the steady-state shear stress value corresponds to the shear strength of the sample.

When comparing the results of our 2D model to the experimental data; slip, slip velocity and shear stress are always represented as average values along the fault line.

### Rate-and-state friction theory

For the characterization of fault reactivation and dynamic slip we use a rate-and-state friction (RSF) formalism in our simulations^29–31^. The rate-and-state constitutive equations describe the evolution of the friction coefficient along the fault as a function of resolved slip rate and the temporal variation in the internal state of the fault surface^29^.

By considering a single internal state variable ($\theta$), the basic equations of RSF read as:

4 $$\mu =f\left(V,\theta \right)$$

5 $$\frac{d\theta }{dt}=g(V,\theta )$$

where $\mu$ is the friction coefficient along the fault given as a function of the slip rate,$V$, and the state variable of the fault, $\theta$. Generally, the state variables can be associated with any parameter suitable for the characterization of the sliding surface, e.g. the average size of the asperity contacts, grain size distribution or porosity of the fault gouge. Dieterich^29^ linked the state variable to the average contact time of the asperities. Given a constant slip velocity, the contact time is proportional to the size distribution of asperities and inversely proportional to the slip rate^32^.

An important observation of velocity-stepping experiments is the so-called “direct effect”, which is incorporated in the rate-and-state laws as the requirement

6 $$\frac{\partial f\left(V,\theta \right)}{\partial V}>0$$

which means that a sudden increase or decrease in slip rate $V$ infers an increasing or decreasing friction coefficient $\mu$, respectively^33^.

The most commonly used explicit form of Eq. (4) is

7 $$\mu \left(V, \theta \right)={\mu }_{0}+a\mathrm\,{ln}\left(\frac{V\left(t\right)}{{V}_{0}}\right)+b\mathrm\,{ln}\left(\frac{{V}_{0}\theta \left(t\right)}{{D}_{c}}\right)$$

where ${\mu }_{0}$ is a nominal coefficient of friction at the reference slip rate ${V}_{0}$; $a$ and $b$ are scaling parameters related to the direct effect and the evolution effect, respectively; and ${D}_{c}$ is the characteristic distance over which the frictional resistance evolves after a sudden change in sliding velocity^32^.

The state evolution law, Eq. (5) was formulated in various ways by several authors. The evolution equation we adopt in this study is the slip law^31^:

8 $$\frac{d\theta (t)}{dt}=-\frac{V\left(t\right)\theta \left(t\right)}{{D}_{c}}ln\left(\frac{V\left(t\right)\theta \left(t\right)}{{D}_{c}}\right)$$

Due to the logarithmic form of Eq. (7), shear stress is not mathematically defined in the case of $V=0$ along the fault, which is the case in the moment before reactivation occurs. Regularized rate-and-state equations were proposed to circumvent this behaviour by Rice and Ben-Zion^34^, so that Eq. (7) can be rewritten as:

9 $$\mu \left(\theta ,V\right)=a \cdot \mathrm{arcsinh}\left[\frac{V(t)}{2{V}_{0}}\mathit{exp}\left(\frac{\theta (t)}{a}\right)\right]$$

and the slip law [Eq. (8)] takes the form of

10 $$\frac{d\theta (t)}{dt}=-\frac{V\left(t\right)}{{D}_{c}}\left(\mu \left(\theta ,V\right)-{\mu }_{ss}\right)$$

where ${\mu }_{ss}={\mu }_{0}+\left(a-b\right)\cdot \mathrm{ln}(V(t)/{V}_{0})$ is the static friction coefficient when $\frac{dV}{dt}=0$.

Here we note that the combined parameter (a–b) determines the slope of the static frictional dependence on the logarithm of the slip velocity. If $a>b$, the slope is positive and the fault is characterized by velocity strengthening friction. If $a<b$, the slope is negative and the fault is in the velocity weakening regime^32^.

Note, that this is different from the frictional weakening and healing of a fault, i.e. the reduction or increase of its friction coefficient, respectively, due to the micro- and macro-mechanical and chemical processes that govern the evolution of the frictional strength of faults over time. Considering the current model of a seismic cycle, frictional healing would encompass all the processes that cause a fault to regain its frictional strength during its inter-seismic evolution after releasing (part of) the accumulated elastic energy, while frictional weakening would indicate the processes that control the loss of frictional strength, which can occur either co-seismically or inter-seismically^16^. In a rate-and-state framework, these processes are integrated into an internal dependency of the fault friction coefficient (and thus the frictional strength) on the sliding velocity and the internal state variable. Mathematically, these dependencies are expressed by the system of Eqs. (9)–(11).

To simulate fault reactivation, the stress balance along the fault is described as

11 $$\tau + \delta {\tau }_{qs} - {\beta }_{rad}V = \mu (\theta ,V) \left({\sigma }_{N}^{^{\prime}} + \delta {\sigma }_{qs}\right)$$

where $\tau$ and ${\sigma }_{N}^{^{\prime}}$ are the shear stress and effective normal stress (i.e. the difference of the normal stress and the pore pressure acting on the fault) induced on the fault due to interaction with the reservoir, $\delta {\tau }_{qs}$ and $\delta {\sigma }_{qs}$ are the quasi-static shear stress and normal stress components corresponding to the elastic stress transfer caused by fault slip. $V$ is slip velocity and ${\beta }_{rad}=G/2{v}_{s}$ is the radiation damping coefficient with $G$ being the shear modulus ($G$ ≈ 11 GPa for Bentheim sandstone)^11^ and ${v}_{s}$ the shear wave velocity (${v}_{s}$≈ 2.3 km/s for Bentheim sandstone)^35^. To obtain the shear displacement, ${u}_{s}$, along the fault, the ordinary differential equation system consisting of Eq. (9), (10) and (11) are solved together (within a Newton–Raphson iterative loop) for the slip velocity vector, which is then used to compute the corresponding value of displacements along the fault. The system of these equations is implemented in a fully-coupled manner in the finite element numerical simulator, GOLEM^36^. The code is built on the object-oriented numerical framework, MOOSE^37^, providing parallel implicit coupling to solve multiphysics problems.

## Supplementary Information

Supplementary Information 1.

Supplementary Information 2.

## Data Availability

The datasets generated and/or analysed during the current study are included in the Supplementary Information files. The laboratory data is available in the supplementary material of Wang et al. (2020a)^11^. The source code of the numerical code GOLEM is hosted on the internal GitLab repository of GFZ and available from the corresponding author on reasonable request.
