# Supplementary material for: Numerical investigation of the effect of fluid pressurization rate on laboratory-scale injection-induced fault slip
Source: Sci Rep. 2023 Mar 17;13:4437. doi: 10.1038/s41598-023-30866-8 (PMC10023684; doi:10.1038/s41598-023-30866-8)
Supplement: Supplementary file 1 — Supplementary Information 1. [file 41598_2023_30866_MOESM1_ESM.pdf]

Supporting information for

Numerical investigation of the effect of fluid pressurization rate on laboratory-scale injection-induced fault slip

Gergő András Hutka[1,2*], Mauro Cacace[3], Hannes Hofmann[1,2], Arno Zang[4,5], Lei Wang[6], Yinlin Ji[1]

[1]Helmholtz Centre Potsdam GFZ German Research Centre for Geosciences, Section 4.8 Geoenergy, Telegrafenberg, 14473 Potsdam, Germany

[2]Institute for Applied Geosciences, Technical University of Berlin, 10587 Berlin, Germany

[3]Helmholtz Centre Potsdam GFZ German Research Centre for Geosciences, Section 4.5 Basin Modelling, Telegrafenberg, 14473 Potsdam, Germany

[4]Helmholtz Centre Potsdam GFZ German Research Centre for Geosciences, Section 2.6 Seismic Hazard and Risk Dynamics, Telegrafenberg, 14473 Potsdam, Germany

[5]Institute of Geosciences, University of Potsdam, Potsdam 14476, Germany

[6]Helmholtz Centre Potsdam GFZ German Research Centre for Geosciences, Section 4.2 Geomechanics and Scientific Drilling, Telegrafenberg, 14473 Potsdam, Germany

*Corresponding author's email: hutka@gfz-potsdam.de, GFZ Section 4.8: Geoenergy

Contents of this file

Supplementary Figures S1-S7

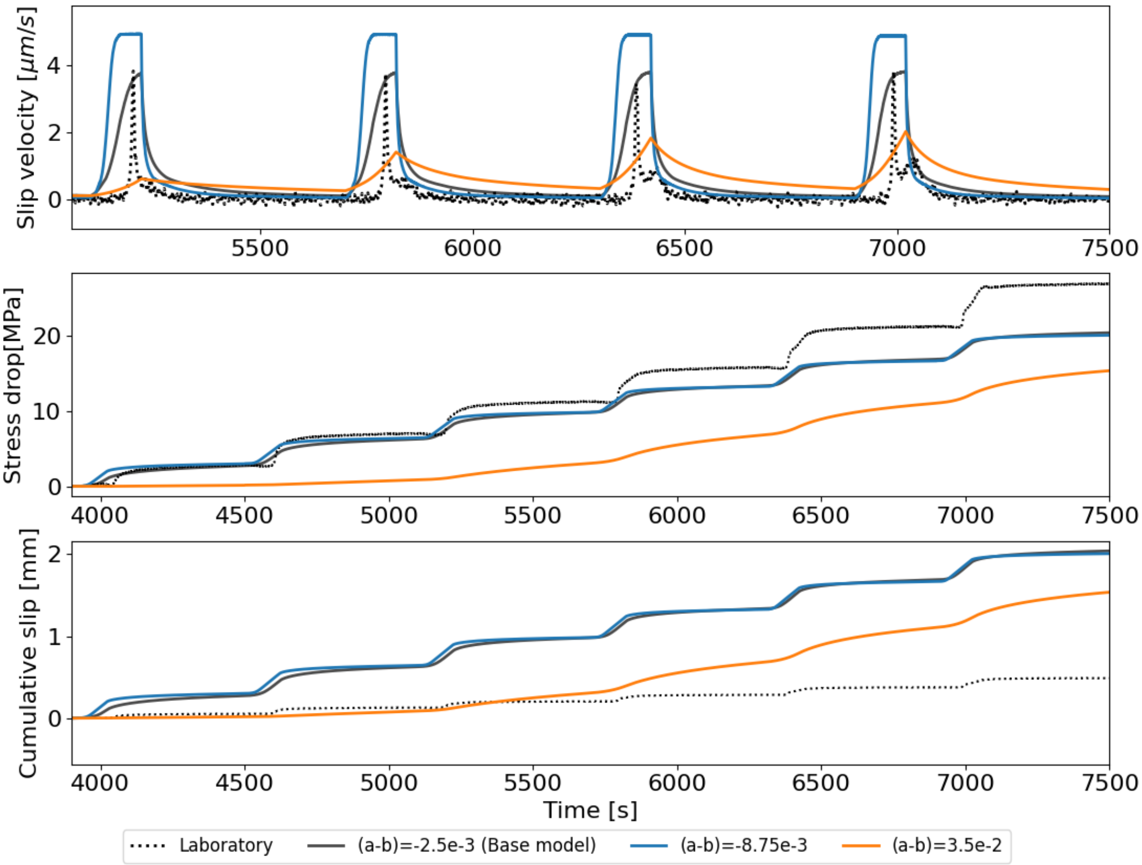

Figure S1. Influence of the rate-and-state parameter *(a-b)* on the slip velocity, stress drop and cumulative slip history compared to the 2 MPa/min laboratory data and base model scenario. Slip velocity is shown from 5050 s for easier interpretation.

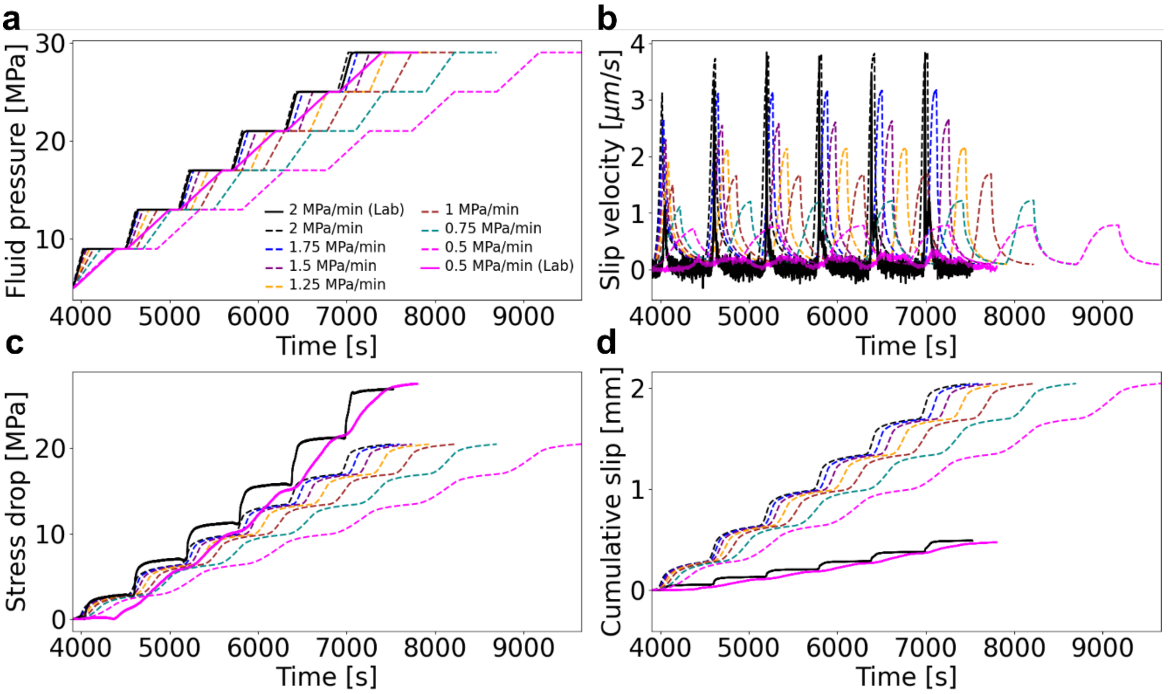

Figure S2. Parametric study on the imposed pressurization rates with fixed, 480 s long constant pressure phases. **a** Pressurization schemes; **b** slip velocity; **c** stress drop; **d** cumulative slip. Dashed lines: simulated curves; solid lines: laboratory measurements.

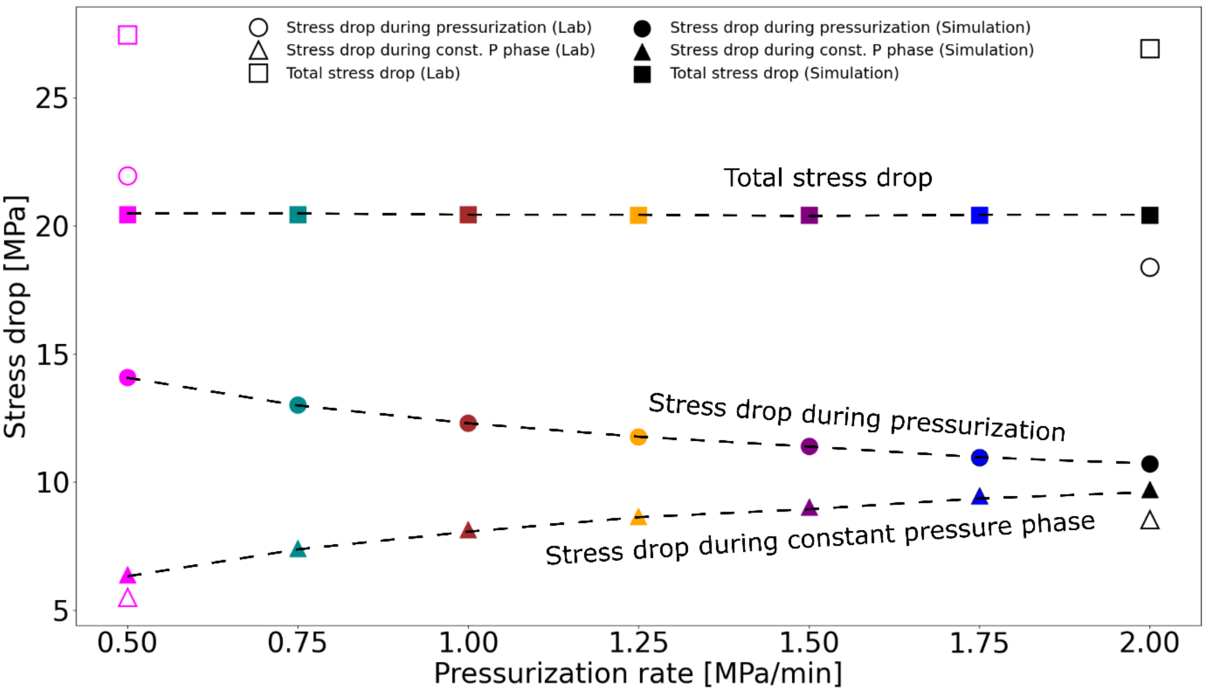

Figure S3. The computed effect of the applied pressurization rate on the shear stress drop (solid symbols) summed over each pressurization period (circles), over each constant pressure period (triangles); and the total stress drop (squares) with fixed, 480 s long constant pressure phases independent of the fluid pressurization rate. Laboratory values are represented by open symbols. The colours represent different pressurization rates and remain the same as in Fig. S2.

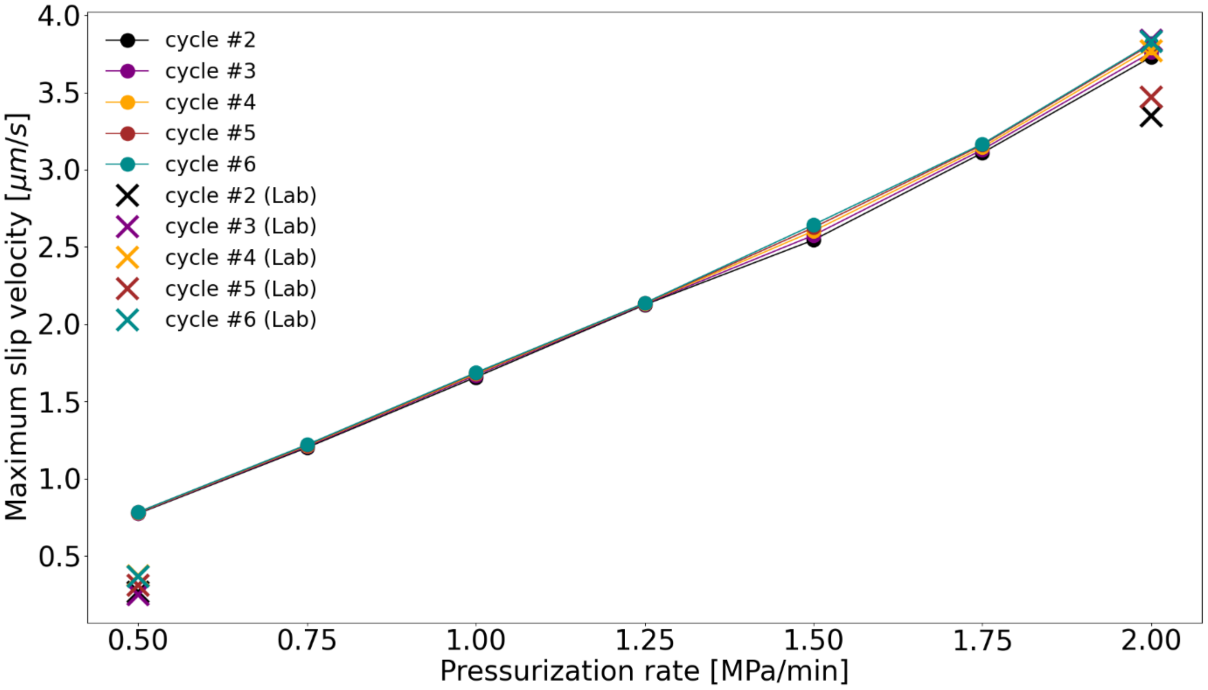

Figure S4. Computed maximum slip velocities versus pressurization rates for stimulation cycles 2 to 6 with fixed, 480 s long constant pressure phases independent of the fluid pressurization rate. Laboratory values are denoted by crosses at 0.5 MPa/min and 2 MPa/min. The first cycle was omitted due to its outlier values (the fluid injection stopped shortly after the onset of fault reactivation in the laboratory test SC1).

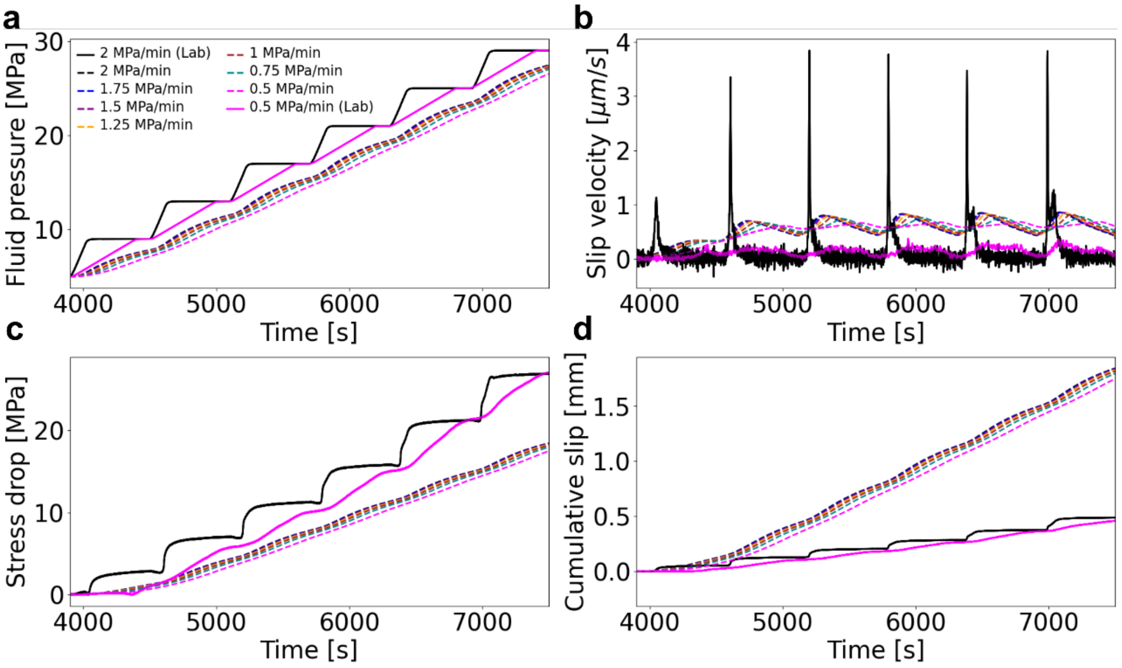

Figure S5. Parametric study on the imposed pressurization rates with a matrix permeability of 1e-6 Darcy. **a** Pressurization schemes; **b** slip velocity; **c** stress drop; **d** cumulative slip. Dashed lines: simulated curves; solid lines: laboratory measurements.

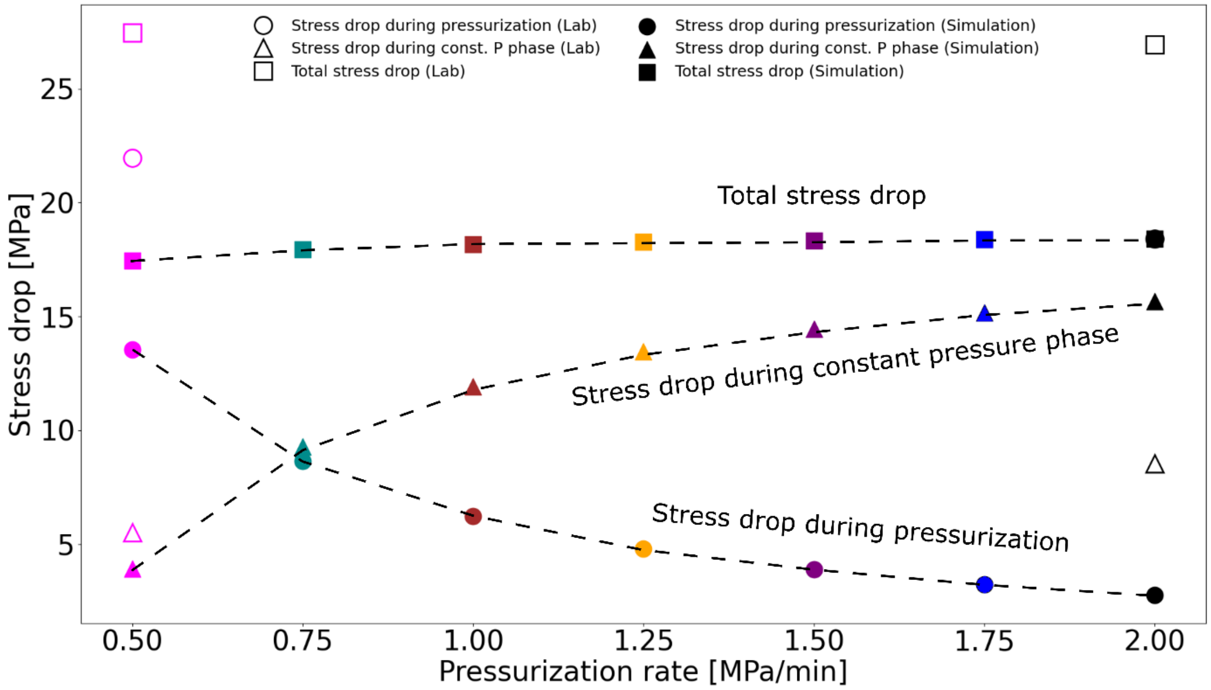

Figure S6. The computed effect of the applied pressurization rate on the shear stress drop (solid symbols) summed over each pressurization period (circles), over each constant pressure period (triangles); and the total stress drop (squares) with a matrix permeability of 1e-6 Darcy. Laboratory values are represented by open symbols. The colours represent different pressurization rates and remain the same as in Fig. S5.

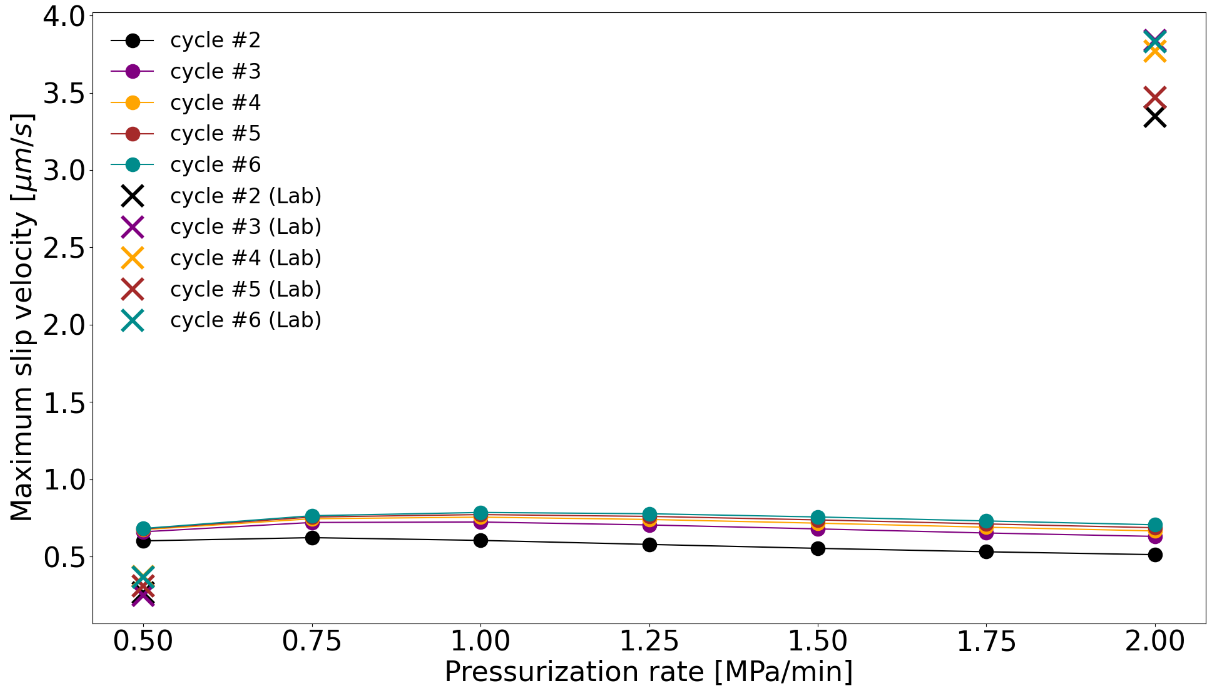

Figure S7. Computed maximum slip velocities versus pressurization rates for stimulation cycles 2 to 6 with a matrix permeability of 1e-6 Darcy. Laboratory values are denoted by crosses at 0.5 MPa/min and 2 MPa/min. The first cycle was omitted due to its outlier values (the fluid injection stopped shortly after the onset of fault reactivation in laboratory test SC1).

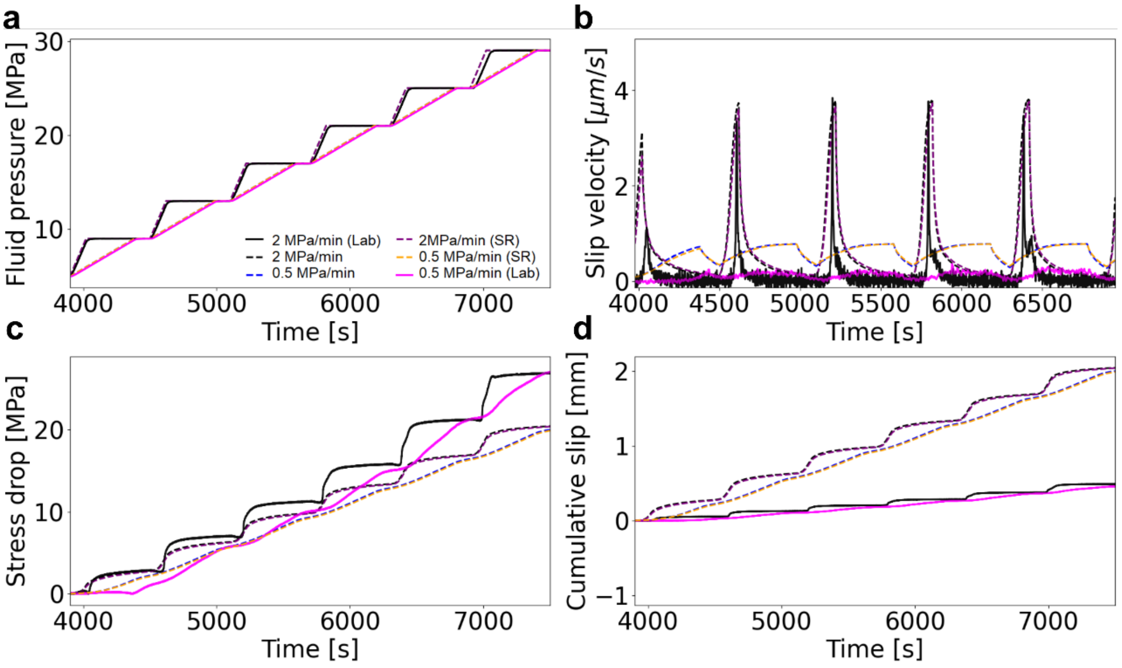

Figure S8. Comparison of the SC1 and SC2 simulations with and without the stressing rate term included in the model. **a** Pressurization schemes; **b** slip velocity; **c** stress drop; **d** cumulative slip. Dashed lines: simulated curves; solid lines: laboratory measurements. SR denotes the models including the stressing rate terms.

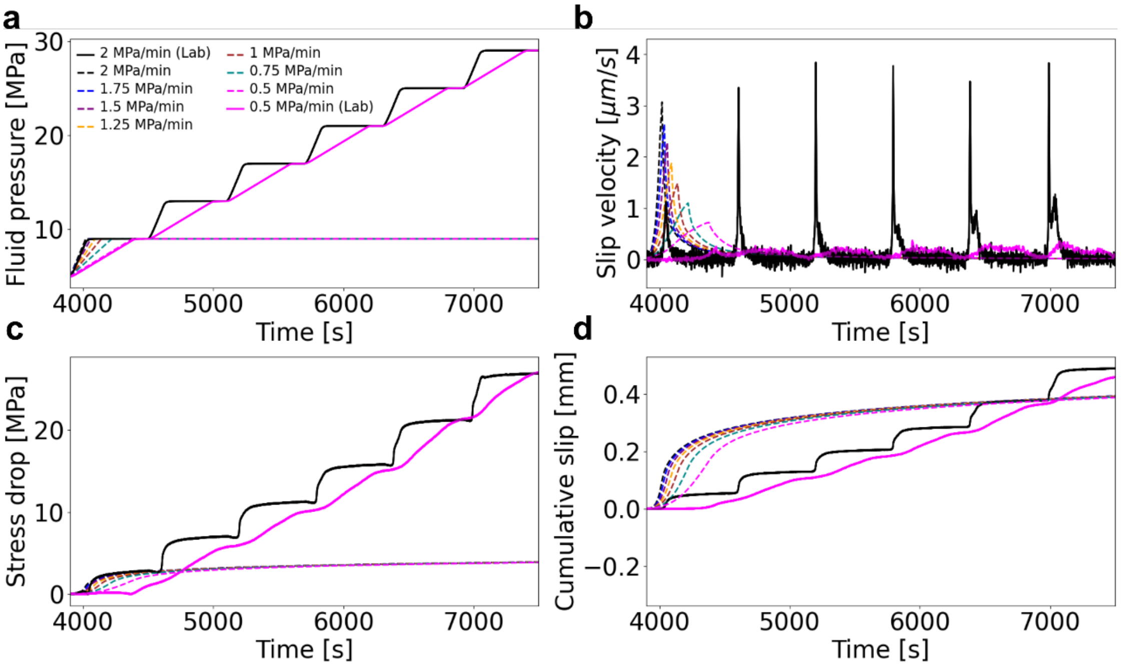

Figure S9. Parametric study on the imposed pressurization rates when the fluid pressure is held at 9 MPa after a single fluid pressurization phase. **a** Pressurization schemes; **b** slip velocity; **c** stress drop; **d** cumulative slip. Dashed lines: simulated curves; solid lines: laboratory measurements.
